# Supplementary material for: Pan-Genomics Reveals a New Variation Pattern of Secreted Proteins in Pyricularia oryzae
Source: J Fungi (Basel). 2022 Nov 23;8(12):1238. doi: 10.3390/jof8121238 (PMC9785059; doi:10.3390/jof8121238)
Supplement: Supplementary file 1 [file jof-08-01238-s001.zip › jof-2027259-supplementary.pdf]

Table S1. The genome assemblies used in this study

Strain[a]	Size (Mb)	Assembly	WGS	Scaffolds/contigs	Level	BUSCO (%)	Cluster[b]	Institute[c]
1106.2	37.56	GCA_000734095.1	JMKE01	5680	Contig	95.4	1	SNU
13FM-16-1	38.39	GCA_003015385.1	MQNR01	1648	Scaffold	96.7	1	FAFU
13FM-24-1	38.57	GCA_003015395.1	MQNS01	1772	Scaffold	96.7	1	FAFU
13FM-3-2	38.87	GCA_003015405.1	MQNT01	1849	Scaffold	96.7	2	FAFU
13FM-5-1	38.48	GCA_003015425.1	MQNU01	1820	Scaffold	96.6	1	FAFU
13FM-9-1	38.81	GCA_003015465.1	MQNV01	1885	Scaffold	96.7	2	FAFU
1801.4	35.76	GCA_000734085.1	JMKD01	8396	Contig	85.5	NA	SNU
1836.3	35.82	GCA_000734105.1	JMKC01	7899	Contig	87.5	NA	SNU
2303.1	38.05	GCA_000734075.1	JMKB01	6787	Contig	95.1	1	SNU
401.4	36.04	GCA_000734165.1	JMJY01	8275	Contig	86.3	NA	SNU
4091-5-8	37.52	GCA_000376765.1	AOCH01	8886	Contig	NA	NA	SNU
4403.2	36.26	GCA_000734155.1	JMKA01	8416	Contig	86.4	NA	SNU
4603.4	37.59	GCA_000734215.1	JMJZ01	5947	Contig	95.3	2	SNU
70-15	40.98	GCA_000002495.2	AACU03	53	Chromosome	96.7	1	other
87-120	39.17	GCA_003013125.1	PQBK01	1985	Scaffold	96.2	1	IWBC
903.4	36.21	GCA_000734185.1	JMJX01	8248	Contig	86.7	NA	SNU
98-06	42.31	GCA_000805855.1	JRBC01	284	Scaffold	96.7	2	other
AH06	37.01	GCA_000734335.1	JMJW01	6061	Contig	95.4	2	SNU
Arcadia	40.84	GCA_002925445.1	PJZD01	1968	Scaffold	95.4	3	IWBC
AV1-1-1	38.89	GCA_003015475.1	MQNW01	1808	Scaffold	96.6	4	FAFU
B157	37.76	GCA_000832285.1	AXDJ01	5735	Contig	95.4	1	other
B2	41.77	GCA_002218465.1	MDUN01	1332	Scaffold	96.2	3	IWBC
B2	42.48	GCA_002925425.1	PJZC01	2486	Scaffold	96.2	3	IWBC
B51	43.05	GCA_002925415.1	PJZB01	2827	Scaffold	96.3	3	IWBC

B71	43.63	GCA_001675625.1	LXOQ01	1787	Scaffold	96.6	3	IWBC
Bd8401	41.27	GCA_002925405.1	PJZA01	2898	Scaffold	96.8	3	IWBC
BdBar16-1	42.08	GCA_001675615.1	LXON01	10105	Scaffold	NA	NA	IWBC
BdJes16-1	41.34	GCA_001675595.1	LXOO01	7154	Scaffold	NA	NA	IWBC
BdMeh16-1	42.96	GCA_001675605.1	LXOP01	4301	Scaffold	96.4	3	IWBC
BJ-90-611	38.42	GCA_003015495.1	MQNX01	1771	Scaffold	96.6	2	FAFU
BJ08-8	38.58	GCA_003015515.1	MQNY01	2008	Scaffold	96.8	2	FAFU
BM1-24	36.24	GCA_000734705.1	JMRG01	9429	Contig	84.1	NA	SNU
Bm88324	41.38	GCA_002925385.1	PJYZ01	1731	Scaffold	96.7	3	IWBC
Br130	40.22	GCA_002925325.1	PJYW01	3120	Scaffold	NA	NA	IWBC
Br7	41.73	GCA_002925335.1	PJYY01	2057	Scaffold	96.4	3	IWBC
Br80	42.47	GCA_002925345.1	PJYX01	1035	Scaffold	96.6	3	IWBC
CA205	36	GCA_000734735.1	JMRH01	9152	Contig	82.5	NA	SNU
CHRF	42.14	GCA_002925295.1	PJYV01	1422	Scaffold	96.6	3	IWBC
CHW	40.38	GCA_002925285.1	PJYU01	1346	Scaffold	96.3	3	IWBC
CQ11	36.26	GCA_000734325.1	JMJV01	8541	Contig	87.7	NA	SNU
DB11-621	38.33	GCA_003015545.1	MQNZ01	2046	Scaffold	96.7	2	FAFU
EI9411	40.31	GCA_001548775.1	LOFC01	1661	Scaffold	96.5	3	FAFU
EI9604	41.6	GCA_001548785.1	LOFD01	1346	Scaffold	96.5	3	FAFU
FH	42.93	GCA_002925225.1	PJYS01	2071	Contig	95.4	3	IWBC
FJ0204804	38.03	GCA_003015555.1	MQOA01	1719	Scaffold	96.8	4	FAFU
FJ11SH-527-1	38.24	GCA_003015565.1	MQOB01	1709	Scaffold	96.7	4	FAFU
FJ11YD-673-1	38.34	GCA_003015595.1	MQOC01	1693	Scaffold	96.8	4	FAFU
FJ12JN-084-3	37.28	GCA_003015625.1	MQOD01	1537	Scaffold	96.7	4	FAFU
FJ13SH05-2	37.75	GCA_003015635.1	MQOE01	1746	Scaffold	96.8	4	FAFU
FJ2000-62A	38.59	GCA_003015645.1	MQOF01	1754	Scaffold	96.8	4	FAFU
FJ2000-69A	38.02	GCA_003015655.1	MQOG01	1601	Scaffold	96.7	4	FAFU
FJ2001-112B	38.69	GCA_003015705.1	MQOH01	1697	Scaffold	96.8	4	FAFU
FJ2003-001A1	38.08	GCA_003015735.1	MQOI01	1675	Scaffold	96.7	4	FAFU

FJ2005113B	38.54	GCA_003015745.1	MQOJ01	1702	Scaffold	96.8	4	FAFU
FJ2006-60A3	37.7	GCA_003015755.1	MQOK01	1729	Scaffold	96.7	4	FAFU
FJ43ZB15-68	37.97	GCA_003015805.1	MQOL01	1599	Scaffold	96.8	2	FAFU
FJ72ZC7-77	37.85	GCA_003015815.1	MQOM01	1533	Scaffold	96.7	4	FAFU
FJ78-JJ	38.45	GCA_003015835.1	MQON01	1757	Scaffold	96.6	2	FAFU
FJ81-JY	38.73	GCA_003015825.1	MQOS01	1895	Scaffold	96.7	2	FAFU
FJ81-MH	38.64	GCA_003015885.1	MQOT01	1972	Scaffold	96.7	2	FAFU
FJ81-SW	38.54	GCA_003016095.1	MQOU01	1894	Scaffold	96.7	2	FAFU
FJ81-ZP	37.96	GCA_003015895.1	MQOV01	1638	Scaffold	96.7	2	FAFU
FJ81221ZB11-14	38.56	GCA_003016105.1	MQOW01	2049	Scaffold	96.7	2	FAFU
FJ81278	38.03	GCA_002368515.1	MQOQ01	1415	Scaffold	96.7	2	FAFU
FJ81278	37.87	GCA_000498135.1	ATNU01	3518	Scaffold	96.7	2	other
FJ81278	43.85	GCA_002368475.1	MQOO01	54	Contig	96.7	2	FAFU
FJ86-CT	38.38	GCA_003015925.1	MQOX01	1894	Scaffold	96.6	2	FAFU
FJ86061ZE3-39	39.06	GCA_003015935.1	MQOY01	1749	Scaffold	96.6	2	FAFU
FJ95054B	38.25	GCA_003015955.1	MQOZ01	1598	Scaffold	96.6	2	FAFU
FJ95085AZB13-116	38.15	GCA_003016115.1	MQPA01	1722	Scaffold	96.9	4	FAFU
FJ98099	38.48	GCA_003015975.1	MQPB01	1722	Scaffold	96.8	4	FAFU
FJ99138	37.5	GCA_003016165.1	MQPC01	1646	Scaffold	96.7	4	FAFU
FJSH0703	38.31	GCA_003016005.1	MQPD01	1843	Scaffold	96.8	4	FAFU
FR13	39.84	GCA_002925215.1	PJYR01	3685	Scaffold	96.6	3	IWBC
FR13	44.41	GCA_900474635.1	UCOG01	74	Contig	96.6	3	other
G17	42.06	GCA_002925205.1	PJYQ01	2593	Scaffold	96.5	3	IWBC
G22	41.42	GCA_002925165.1	PJYP01	2453	Scaffold	96.6	3	IWBC
GD-05-029b	37.94	GCA_003016175.1	MQPE01	1653	Scaffold	96.8	4	FAFU
GD06-53	37.4	GCA_003016185.1	MQPF01	1923	Scaffold	96.8	4	FAFU

GD08-2025	37.97	GCA_003016195.1	MQPG01	2008	Scaffold	96.8	4	FAFU
GD22	36.98	GCA_000734345.1	JMJU01	7027	Contig	90.8	NA	SNU
GG11	42.56	GCA_002925155.1	PJYO01	1462	Scaffold	96.4	3	IWBC
GOV41	35.95	GCA_000734675.1	JMRD01	8535	Contig	86.3	NA	SNU
GrF52	39.9	GCA_002925145.1	PJYN01	3580	Scaffold	96.7	3	IWBC
Guy11	37.36	GCA_002925095.1	PJYM01	3318	Scaffold	NA	NA	IWBC
GUY11	37.49	GCA_002368525.1	MQOR01	1182	Scaffold	NA	NA	FAFU
GUY11	42.87	GCA_002368485.1	MQOP01	56	Contig	96.7	1	FAFU
GX01	36.79	GCA_000734395.1	JMJT01	8371	Contig	90.4	NA	SNU
H08-1a	39.38	GCA_002218345.1	NKQF01	2695	Contig	96.9	4	other
H08-1c	39.27	GCA_002218355.1	NKQG01	2879	Scaffold	96.8	4	other
HB-14	38.2	GCA_003016015.1	MQPH01	2005	Scaffold	96.7	1	FAFU
HB-LTH18	38.18	GCA_003016025.1	MQPI01	1601	Scaffold	96.7	1	FAFU
HB12	36.72	GCA_000734235.1	JMJS01	7622	Contig	90.3	NA	SNU
HN-0812-3	37.99	GCA_003016035.1	MQPJ01	1712	Scaffold	96.7	2	FAFU
HN-158	38.11	GCA_003016255.1	MQPK01	1996	Scaffold	96.7	4	FAFU
HN06	36.11	GCA_000734405.1	JMJR01	7798	Contig	88.8	NA	SNU
HN10-1604	37.95	GCA_003016245.1	MQPL01	1957	Scaffold	96.8	4	FAFU
HN19311	36.66	GCA_000475075.1	ATNT01	2998	Scaffold	96.5	1	other
HO	42.98	GCA_002925105.1	PJYL01	1736	Scaffold	96.6	3	IWBC
IA1	39.42	GCA_002925085.1	PJYK01	2230	Scaffold	96.6	2	IWBC
IB33	40.24	GCA_002925065.1	PJYJ01	2752	Scaffold	96.7	1	IWBC
IB49	39.44	GCA_002925045.1	PJYI01	1648	Scaffold	96.7	1	IWBC
IC17	39.53	GCA_002925025.1	PJYH01	1981	Scaffold	96.7	1	IWBC
IE1K	39.6	GCA_002924985.1	PJYG01	1711	Scaffold	96.7	1	IWBC
IT10	36.26	GCA_000734685.1	JMRE01	8855	Contig	85.9	NA	SNU
JL10	36.32	GCA_000734425.1	JMJQ01	8649	Contig	86.8	NA	SNU
JS-10-6-1-2	38.56	GCA_003016085.1	MQPM01	1667	Scaffold	96.7	2	FAFU
JS08-611	38.2	GCA_003016275.1	MQPN01	1976	Scaffold	96.6	2	FAFU

JS09-138	38.47	GCA_003016265.1	MQPO01	2100	Scaffold	96.8	2	FAFU
JS25	36.86	GCA_000734245.1	JMJP01	6159	Contig	95.2	2	SNU
JX-09Z116-1	37.6	GCA_003016325.1	MQPP01	1589	Scaffold	96.8	2	FAFU
JX10-102	37.64	GCA_003016665.1	MQPQ01	1959	Scaffold	96.8	2	FAFU
JX11-141	38.31	GCA_003016655.1	MQPR01	2092	Scaffold	96.8	2	FAFU
K84-01	36.39	GCA_000734455.1	JMQS01	8119	Contig	88.4	NA	SNU
K88-07	35.57	GCA_000734575.1	JMQT01	7624	Contig	89.2	NA	SNU
K88-24	36.29	GCA_000734495.1	JMQU01	7725	Contig	89.9	NA	SNU
K91-10	36.38	GCA_000734595.1	JMQV01	7786	Contig	88.6	NA	SNU
K91-13	36.45	GCA_000734515.1	JMQW01	7603	Contig	90.2	NA	SNU
K91-30	35.67	GCA_000734525.1	JMQX01	9213	Contig	82.6	NA	SNU
K93-16	35.97	GCA_000734605.1	JMQY01	8881	Contig	84	NA	SNU
K96-07	35.61	GCA_000734555.1	JMQZ01	9452	Contig	81.6	NA	SNU
K96-11	35.77	GCA_000734635.1	JMRA01	9202	Contig	82.5	NA	SNU
K98-02	36	GCA_000734755.1	JMRB01	9277	Contig	82.9	NA	SNU
K98-10	37.1	GCA_000734655.1	JMRC01	8594	Contig	90	NA	SNU
KA1-3-1	39.35	GCA_003016705.1	MQPS01	2079	Scaffold	96.8	4	FAFU
KA2-1-1	39.29	GCA_003016715.1	MQPT01	1855	Scaffold	96.7	1	FAFU
KJ201	45.1	GCA_000376685.2	ANSL02	123	Scaffold	95.5	2	SNU
LpKY97	42.91	GCA_002924975.1	PJYF01	1340	Scaffold	96.3	3	IWBC
MG01	39	GCA_000969745.1	AYPX01	7722	Contig	95	1	other
MG02	39.34	GCA_001936075.1	LNTH01	8554	Contig	95.3	1	other
MG04	39.88	GCA_001936935.1	LNTK01	41689	Contig	NA	NA	other
MG10	38.75	GCA_001936435.1	LNTM01	7631	Contig	96.1	4	other
ML33	39.49	GCA_002924965.1	PJYE01	3431	Scaffold	96.7	4	IWBC
Mo-nwi-55	37.76	GCA_002021675.1	AZSW01	7002	Contig	91.2	NA	other
Nich-2-3-2	39.12	GCA_003016385.1	MQPU01	2154	Scaffold	96.6	1	FAFU
Nich-2-7-4	39.16	GCA_003016725.1	MQPV01	2037	Scaffold	96.6	1	FAFU
NX37	35.87	GCA_000734265.1	JMJO01	8518	Contig	86.1	NA	SNU

P-0028	42.99	GCA_002218475.1	MKZV01	1038	Scaffold	96.6	3	IWBC
P-0029	42.45	GCA_002218485.1	MLCC01	818	Scaffold	95.6	3	IWBC
P131	37.96	GCA_000292605.1	AHZT01	1822	Scaffold	96.3	2	other
P28	43.58	GCA_002924945.1	PJYD01	1657	Scaffold	96.6	3	IWBC
P29	43.27	GCA_002924915.1	PJYC01	1468	Scaffold	96.5	3	IWBC
P3	42.62	GCA_002924885.1	PJYB01	2473	Scaffold	96.6	3	IWBC
Pg1213-22	42.08	GCA_002924875.1	PJYA01	1913	Scaffold	96.6	3	IWBC
PH42	43.29	GCA_002924865.1	PJXZ01	2757	Scaffold	95	3	IWBC
PL2-1	42.55	GCA_002924835.1	PJXY01	811	Scaffold	96.5	3	IWBC
PL3-1	42.48	GCA_002924825.1	PJXX01	1722	Contig	96.4	3	IWBC
PR72	35.8	GCA_000734785.1	JMRF01	8427	Contig	85.6	NA	SNU
Py22.1	42.44	GCA_002218425.1	MILZ01	900	Scaffold	96.4	3	IWBC
Py5020	42.67	GCA_002218435.1	MKIG01	1386	Scaffold	95.3	3	IWBC
RMg-Dl	34.82	GCA_001853415.2	MBSD02	996	Scaffold	93.3	NA	other
Sar-2-20-1	39.28	GCA_003016745.1	MQPW01	2277	Scaffold	96.6	4	FAFU
Sar-AD3-5	38.99	GCA_003016785.1	MQPX01	1807	Scaffold	96.7	1	FAFU
SC-10-120-65-2	38.33	GCA_003016395.1	MQPY01	1726	Scaffold	96.8	4	FAFU
SC-10-25-44-1	38.72	GCA_003016795.1	MQPZ01	1798	Scaffold	96.7	4	FAFU
SC05	36.68	GCA_000734275.1	JMJN01	8190	Contig	89.1	NA	SNU
SSFL02-1	40.5	GCA_002924795.1	PJXW01	2106	Scaffold	96.4	3	IWBC
SSFL14-3	42.71	GCA_002924785.1	PJXV01	2635	Scaffold	96.3	3	IWBC
SV9610	39.33	GCA_001548845.1	LOFE01	1546	Scaffold	96.6	3	FAFU
SV9623	39.38	GCA_001548855.1	LOFF01	1514	Scaffold	96.5	3	FAFU
T25	42.29	GCA_002924745.1	PJXU01	1276	Scaffold	96.4	3	IWBC
TF05-1	43.54	GCA_002924755.1	PJXT01	1717	Scaffold	96.5	3	IWBC
TW-1-1-1-B-1	39.26	GCA_003016415.1	MQQA01	1976	Scaffold	96.7	2	FAFU
TW-12CY-TB 1-2	38.97	GCA_003016805.1	MQQB01	1864	Scaffold	96.8	2	FAFU

TW-12HL-DF 1-2	38.7	GCA_003016825.1	MQQC01	1802	Scaffold	96.8	2	FAFU
TW-12HL-YL 2-1	38.99	GCA_003016855.1	MQQD01	1920	Scaffold	96.6	2	FAFU
TW-12TD-RH 1-1	39.03	GCA_003016875.1	MQQE01	1791	Scaffold	96.8	2	FAFU
TW-12TN-HB 2-2	39.03	GCA_003016895.1	MQQF01	1805	Scaffold	96.7	2	FAFU
TW-12YL-DL 3-2	38.67	GCA_003016905.1	MQQG01	1859	Scaffold	96.7	2	FAFU
TW-12YL-DP 1-1	38.88	GCA_003016425.1	MQQH01	1927	Scaffold	96.6	2	FAFU
TW-12YL-TT 4-1	38.46	GCA_003016935.1	MQQI01	2175	Scaffold	96.7	2	FAFU
TW-2-7-2-A-1	39.23	GCA_003016465.1	MQQJ01	1861	Scaffold	96.7	2	FAFU
TW-6-2-2-B-1	38.75	GCA_003016955.1	MQQK01	1715	Scaffold	96.9	2	FAFU
TW-6-43-1	39.03	GCA_003016475.1	MQQL01	1860	Scaffold	96.6	2	FAFU
TW-CYBP1-3	39.08	GCA_003016495.1	MQQM01	1850	Scaffold	96.7	2	FAFU
TW-PT1-1	38.93	GCA_003016965.1	MQQN01	1891	Scaffold	96.7	2	FAFU
TW-PT3-1	38.98	GCA_003016505.1	MQQO01	1955	Scaffold	96.7	2	FAFU
TW-PT6-1	39.07	GCA_003016985.1	MQQP01	1894	Scaffold	96.8	2	FAFU
TW-TN4-2	39.2	GCA_003017025.1	MQQQ01	1886	Scaffold	96.8	2	FAFU
V86010	38.93	GCA_002105295.1	MWIT01	1924	Scaffold	95.6	4	FAFU
WBKY11	43.36	GCA_002924685.1	PJXR01	114	Scaffold	96.5	3	IWBC
WBSS	39.24	GCA_002924695.1	PJXQ01	22748	Scaffold	NA	NA	IWBC
WD-3-1_1	38.65	GCA_003017035.1	MQQR01	1877	Scaffold	96.7	4	FAFU
WHTQ	42.48	GCA_002924665.1	PJXP01	3811	Scaffold	NA	NA	IWBC
Y34	38.87	GCA_000292585.1	AHZS01	1198	Scaffold	96.3	2	other
YN07205e	38.75	GCA_003016545.1	MQQS01	2052	Scaffold	96.8	4	FAFU
YN072310	39.3	GCA_003016555.1	MQQT01	2213	Scaffold	96.8	2	FAFU

YN072311	39.25	GCA_003017045.1	MQQU01	2185	Scaffold	96.7	2	FAFU
YN072313	38.8	GCA_003017065.1	MQQV01	1869	Scaffold	96.7	1	FAFU
YN08181e	38.82	GCA_003017115.1	MQQW01	1812	Scaffold	96.8	1	FAFU
YN08182c	39.31	GCA_003017125.1	MQQX01	2562	Scaffold	96.8	1	FAFU
YN126311	39.31	GCA_003017175.1	MQQY01	2199	Scaffold	96.8	2	FAFU
YN126441	38.72	GCA_003016585.1	MQQZ01	1959	Scaffold	96.6	2	FAFU
YN8773-19	37.25	GCA_003017165.1	MQRA01	1498	Scaffold	96.8	1	FAFU
YN8773R-27	37.38	GCA_003016575.1	MQRB01	1548	Scaffold	96.8	1	FAFU
ZJ00-10	37.85	GCA_003017255.1	MQRC01	1875	Scaffold	96.7	2	FAFU
ZJ08-41	37.82	GCA_003016625.1	MQRD01	1871	Scaffold	96.7	2	FAFU
ZJ15	37.49	GCA_000734315.1	JMJM01	6575	Contig	95.7	2	SNU
ZJ2011-7-1	38.62	GCA_003016635.1	MQRE01	1663	Scaffold	96.8	2	FAFU

[a]Genomes with a completeness BUSCO <95% were not included in this study；

[b]SNU, Seoul National University; IWBC, International Wheat Blast Consortium; FAFU, Fujian Agriculture and Forestry University;

[c]Cluster 1, 2, 3 and 4 indicate the four Clade of *P. oryzae* grouped according to the PAV of selected genes.
